# Identifying risk factors for readmission to the ICU: a qualitative approach

**DOI:** 10.1186/cc12409

**Published:** 2013-03-19

**Authors:** DG Strange, K Tjelle, A Lindhardt, K Jensen

**Affiliations:** 1 Bispebjerg Hospital, Copenhagen NV, Denmark

## Introduction

Readmission to the ICU within 48 hours is an indicator of quality of intensive care and is associated with an increase in mortality. During the last years several groups have published data based on multivariate logistic regression analysis to describe characteristics of patients who needed readmission to the ICU. Older age, comorbid conditions and severity of illness (APACHE score) have been among the strongest predictors for readmission. In our ICU most patients are in the groups formerly identified as risk groups, which means that stratification and prediction of readmission is difficult. Because of the unusual high severity of acute and pre-existing illnesses we could not find a data match on comparable patient groups. To investigate whether we could reduce our rate of readmission we therefore decided to perform a qualitative investigation to identify risk factors related to readmission. After identification of the risk factors we will take actions to optimize care and perform ongoing control of the implemented actions to secure that they decreases the rate of readmission.

## Methods

Retrospective data on patients readmitted to the ICU within 48 hours during an 8-month period (November 2011 to June 2012) were drawn from the Critical Information System (CIS) at ICU ZIT, Bispebjerg Hospital, Denmark. ZIT is a multidisciplinary unit with 10 beds and 500 to 600 admissions/year and a median SAPS II score of 47. A group of consultants, junior doctors and nurses from the ICU and the ward each read the patient files with focus on pattern recognition and suggested trigger points to focus on. Data on trigger points were then drawn from the CIS system and re-evaluated. Finally, five key points were identified and serves as basis for future actions.

## Results

In a qualitative analysis, readmissions to the ICU are related to the following five key points - discharge outside days hours, lack of infection control, stay in ICU <2 days, lung physiotherapy ordinated but not effectuated, and several minor organ dysfunctions (atrial fibrillation and acute kidney injury). Age, diagnosis, SAPS II score or ventilator treatment during intensive care was not different in patients with successful discharge and patients readmitted in this group of patients.

## Conclusion

It is possible and suitable to identify key points for future efforts in a given subgroup of patients using a systematic qualitative approach.

